# Children's eating behavior, feeding practices of parents and weight problems in early childhood: results from the population-based Generation R Study

**DOI:** 10.1186/1479-5868-9-130

**Published:** 2012-10-30

**Authors:** Pauline W Jansen, Sabine J Roza, Vincent WV Jaddoe, Joreintje D Mackenbach, Hein Raat, Albert Hofman, Frank C Verhulst, Henning Tiemeier

**Affiliations:** 1 The Generation R Study Group, Erasmus MC-University Medical Center Rotterdam, Rotterdam, the Netherlands; 2 Department of Child & Adolescent Psychiatry / Psychology, Erasmus MC-University Medical Center Rotterdam, PO-BOX 2060, Rotterdam, 3000 CB, The Netherlands; 3 Department of Psychiatry, Erasmus MC-University Medical Center Rotterdam, Rotterdam, the Netherlands; 4 Department of Epidemiology, Erasmus MC-University Medical Center Rotterdam, Rotterdam, the Netherlands; 5 Department of Pediatrics, Erasmus MC-University Medical Center Rotterdam, Rotterdam, the Netherlands; 6 Department of Public Health, Erasmus MC-University Medical Center Rotterdam, Rotterdam, the Netherlands

**Keywords:** Overweight, Underweight, BMI, Eating behavior, Feeding, Parenting, Children

## Abstract

**Background:**

Weight problems that arise in the first years of life tend to persist. Behavioral research in this period can provide information on the modifiable etiology of unhealthy weight. The present study aimed to replicate findings from previous small-scale studies by examining whether different aspects of preschooler’s eating behavior and parental feeding practices are associated with body mass index (BMI) and weight status -including underweight, overweight and obesity- in a population sample of preschool children.

**Methods:**

Cross-sectional data on the Child Eating Behaviour Questionnaire, Child Feeding Questionnaire and objectively measured BMI was available for 4987 four-year-olds participating in a population-based cohort in the Netherlands.

**Results:**

Thirteen percent of the preschoolers had underweight, 8*%* overweight, and 2*%* obesity. Higher levels of children’s Food Responsiveness, Enjoyment of Food and parental Restriction were associated with a higher mean BMI independent of measured confounders. Emotional Undereating, Satiety Responsiveness and Fussiness of children as well as parents’ Pressure to Eat were negatively related with children’s BMI. Similar trends were found with BMI categorized into underweight, normal weight, overweight and obesity. Part of the association between children’s eating behaviors and BMI was accounted for by parental feeding practices (changes in effect estimates: 20-43%), while children’s eating behaviors in turn explained part of the relation between parental feeding and child BMI (changes in effect estimates: 33-47%).

**Conclusions:**

This study provides important information by showing how young children’s eating behaviors and parental feeding patterns differ between children with normal weight, underweight and overweight. The high prevalence of under- and overweight among preschoolers suggest prevention interventions targeting unhealthy weights should start early in life. Although longitudinal studies are necessary to ascertain causal directions, efforts to prevent or treat unhealthy child weight might benefit from a focus on changing the behaviors of both children and their parents.

## Background

Weight problems in childhood and adolescence are very common in Western countries with overweight rates estimated at 17-25% in West-Europe, Australia, and the United States [1-3]. Underweight is less prevalent than overweight but an estimated 3-8% of children in developed countries have underweight [4,5]. Childhood under- and overweight are an important public health problem, as these conditions tend to have a chronic character (underweight [6]; overweight [7,8]) and predict a wide range of future morbidity. Overweight in children is associated with future cardiovascular diseases [8,9], diabetes [10], and psychosocial problems [7,11]. Furthermore, even though thinness is nowadays likely to represent the lower end of the healthy weight distribution [12], there is also evidence that a low body mass index (BMI) in early childhood is a risk factor for later coronary heart disease [9] in Western populations.

Against the background of the common occurrence and chronic course of childhood weight problems it is important to advance our knowledge of their etiology. Several risk factors for childhood overweight have been identified, such as parental weight status, early growth and children’s physical activity and sedentary behavior, with some of these risk factors seemingly easier modifiable than others [13,14]. Behavioral research also provides information on the modifiable etiology of weight problems and suggested that children’s eating behavior and appetite-related traits are associated with BMI [15,16]. Moreover, it has been shown that parents exert an important influence on children’s eating patterns and weight development through their own eating behaviors and feeding practices [17,18]. This evidence is mainly based on studies in school-aged children and adolescents [15-18], while it has been argued that weight problems can already arise earlier in life [19,20]. The first few years of life are characterized by rapid growth and encompass several critical periods in children’s growth trajectories [21]. Moreover, young children go through remarkable transitions in digestive behavior, and evidence points to children’s eating behaviors being established by the end of the preschool period and remaining stable thereafter [22]. This makes preschool children a particularly important target group for interventions aimed at enhancing healthy eating behaviors and a healthy weight. Given the stability of eating behaviors and appetitive traits across early and later childhood [22-24], we hypothesized that the relation between eating behavior and weight status does not differ substantially between the different age groups. However, there is a need to confirm associations among young children before evidence-based interventions aimed at the prevention and worsening of unhealthy child weight can be implemented.

Considering the importance of the early developmental period, researchers started attempting to identify behavioral influences on BMI in preschoolers such as parental feeding practices. Parental pressure on children to eat was negatively related to child BMI [25-28], while parents’ restrictions regarding food intake was positively associated with children’s weight [27,29]. This behavior of parents can be a response to children’s weight status, but parental feeding practices also elicit certain child eating behaviors that in turn may influence weight development. However, several other studies have not been able to replicate these findings [25,26,28,30,31]. Moreover, other dimensions of parental feeding, like control and monitoring, were hardly associated with preschoolers’ BMI [25-28,30-33].

Eating behaviors of young children, such as eating in response to environmental food cues, increase the likelihood of children to have a high BMI [25,29], while responsiveness to internal satiety cues and pickiness have been associated with a lower mean BMI [15,34]. Again, these associations were not consistently found [25,26,35,36]. The lack of findings between children’s eating behaviors and BMI, but also between parental feeding practices and child BMI, could well be due to limited statistical power. In contrast to several studies examining these associations in older children e.g., [16,37,38] research in preschool children was mainly conducted in small samples including less than 300 children [25-27,29-33,35,36], with a few exceptions [15,28,34]. Moreover, research in this field was hampered by the use of high-risk groups, such as children at risk of overweight or from low-income families [26,27,32,33,35]. This limits generalizibility of results, as associations might be different at the population level. Findings of several earlier studies should also be interpreted with caution as parent reports of children’s anthropometrics were used [15,25,35,36]. Parents tend to underestimate their child’s weight, especially if the child is overweight or obese [39], and many children with overweight might be missed. Finally, although several studies had information available on both children’s eating behaviors and parental feeding practices [25,26,29,35], it remains largely unknown if eating behavior of children and feeding behavior of parents have an independent effect on child BMI or whether these behaviors partly account for each others effect on child BMI. There is some evidence for the latter: Joyce and colleagues showed that preschool children’s disinhibited eating – a composite score of food responsiveness and emotional overeating – partially mediated the association between parental restriction and children’s BMI [29]. Research in school-aged children also suggests complex associations between parental behavior, and children’s eating and BMI [40,41]. These studies hypothesized a child-responsive model postulating that child characteristics influence parental behavior [41].

The present study aims to replicate findings of previous behavioral studies in preschool children by examining whether young children’s eating behavior and parental feeding practices are associated with objectively measured BMI in a large population-based cohort of four-year olds. Different food approach and food avoidant behaviors of children, as well as three different parenting dimensions will be examined. Moreover, not only overweight and obesity, but underweight will be studied as well. Childhood underweight is a highly understudied area of behavioral research despite the evidence that this condition is a risk factor for future morbidity just like overweight [9]. Based on previous studies, we hypothesized that children with high levels of food *approach* behaviors like food responsiveness have a higher mean BMI, and that food *avoidant* behaviors such as satiety responsiveness and fussiness are associated with a lower mean BMI. Consistent with a child-responsive model [41], we also expected that parents of children with overweight or high levels of food approach behaviors are more restrictive. These parents would also exert less pressure on their children to eat than parents of children with a normal weight or with high levels of food avoidant behaviors. Finally, we hypothesized that eating behavior of children is associated with BMI independently of parental feeding practices. In accordance with a child-responsive model [41], we also expected that the relation between parental feeding and child BMI is fully explained by children’s eating behaviors.

## Methods

### Design and study population

This study was embedded in Generation R, a population-based cohort from fetal life onwards [42]. Briefly, all pregnant women living in Rotterdam, the Netherlands, with an expected delivery date between April 2002 and January 2006 were invited to participate (participation rate: 61%). The ethnic distribution of participants differed only moderately from that of the population in the study area [42]. However, mean household income and educational attainment were slightly higher among study participants. Written informed consent was obtained from all participants. The Medical Ethical Committee of the Erasmus Medical Center, Rotterdam, has approved the study. Information of the participants was obtained by postal questionnaires filled out by parents, and from medical records of hospitals, midwives, and Child Health Centers. Full consent for the postnatal phase of the Generation R Study was obtained from 7295 children and their parents. For 4987 of these children, data on at least one of the subscales of eating behavior was available. The population per analyses varied slightly per subscale due to missing data on subscales (*n* between 4911 and 4967). Information on BMI was available for 3157 children.

Comparison of non-responders (*n* = 2308) and responders (*n* = 4987) indicated that data on eating behavior was more often missing in children of non-Dutch origin, *χ*^2^(1, 6738) = 414, *p* < .001, with a higher BMI, *F*(1, 4206) = 11, *p* = .001, with lower educated mothers, *χ*^2^(1, 6557) = 497, *p* < .001, and higher maternal BMI, *F*(1, 6521) = 55, *p* < .001, as compared to children with complete data on eating behavior. In contrast, among the children with available data on eating behavior (*n* = 4987), children with (*n* = 3157) and without (*n* = 1828) data on BMI did not differ from each other with respect to national origin, *χ*^2^(1, 4803) = 3, *p* = .085, maternal educational level, *χ*^2^(1, 4715) = 0.1, *p* = .724, or maternal BMI, *F*(1, 4440) = 0.08, *p* = .782, suggesting that data on BMI was missing at random.

### Children’s eating behavior

Eating behavior was assessed by postal questionnaire including the Child Eating Behaviour Questionnaire (CEBQ) and Child Feeding Questionnaire (CFQ). Parents were asked to fill out these questionnaires around the fourth birthday of their child. The translation of the original English questionnaires was carried out using a standard forward-backward translation method [43].

The CEBQ [44] is a 35-item instrument designed to assess variation in eating style among children. The CEBQ consists of seven subscales, four of which measure food approach behaviors: Emotional Overeating, Enjoyment of Food, Food Responsiveness, and Desire to Drink. The other three subscales quantify food-avoidant behavior: Emotional Undereating, Satiety Responsiveness, and Fussiness. Examples of items are “My child loves food” (Enjoyment of Food), “Even if my child is full up, s/he finds room to eat his/her favorite food” (Food Responsiveness), “My child eats less when upset” (Emotional Undereating), and “My child has a big appetite” (Satiety Responsiveness). The CEBQ has good psychometric properties, such as good internal consistency, concurrent validity with actual eating behavior, test-retest reliability, and stability over time [34,44-46]. Good internal consistency was confirmed in our sample with Cronbach alpha’s ranging from .78 to .89.

Three subscales of the CFQ [37] were used to assess parental attitudes and strategies regarding control of children’s eating: Monitoring (3 items), Restriction (8 items), and Pressure to Eat (4 items). Examples of items are “How much do you keep track of the high fat foods your child eats?” (Monitoring), and “I intentionally keep some foods out of my child’s reach” (Restriction). Research provided evidence for concurrent validity of the CFQ with actual observations of feeding behavior of mothers [47]. Furthermore, the CFQ-scales correlate well with children’s actual food intake [48] and children’s BMI in two small scale samples [37] indicating high external validity. Reliability of the administered CFQ-scales was moderate (*α* = .66, Pressure to Eat) to high (*α* = .92, Monitoring).

CEBQ and CFQ items were answered on a five-point Likert scale from 1=never to 5=always. Scale scores were only calculated if at least 75*%* of the items were completed. The continuous CEBQ and CFQ scale scores were expressed as standard deviation scores to facilitate effect size comparison between scales.

### BMI

Trained staff of the municipal Child Health Centers obtained children’s growth characteristics as part of a routine health care program. Children visit the centers on a regular basis and the present study uses data from the visit scheduled around the fourth birthday. Weight was measured by a mechanical personal scale (SECA®) while children were wearing underwear only. Height was measured in standing position by a Harpenden stadiometer (Holtain Limited®). Body Mass Index (BMI) was calculated as weight/height^2^ (kg/m^2^). Age- and sex-specific BMI standard deviation scores were calculated using the Dutch reference [49] in the Growth Analyzer program (http://www.growthanalyser.org). International age- and sex-specific cut offs were used to classify children into four different weight groups: underweight [50], normal weight, overweight and obesity [51].

### Covariates

Several child and parental characteristics were considered as possible confounders, as they were previously linked with children’s BMI and eating behaviors [14,47,52]. Information about child gender, date of birth (to calculate age), and birth weight were obtained from midwife and hospital registries. National origin of the child was based on country of birth of both parents, as assessed by parental questionnaire. Mothers also reported in postal questionnaires about educational level, family income, smoking habits during pregnancy and global psychopathology, which was assessed with the Brief Symptom Inventory [53]. Paternal psychopathology was assessed using the same instrument in a separate questionnaire filled out by the fathers. Height and weight were measured in mothers and fathers at the Generation R research centre. Parental BMI was calculated as weight/height^2^.

### Statistical analyses

The distribution of confounders is presented stratified by weight status. The *χ*^*2*^-statistic was used to test whether the distribution of categorical covariates differed between the weight categories; ANOVA’s were used for continuous covariates, and Kruskal-Wallis tests for continuous non-normally distributed covariates. The association between the CEBQ, CFQ and child weight was first explored with Pearson’s correlation coefficients. Effect sizes of Pearson’s correlation are interpreted as small for *r* around 0.10, medium for *r* around 0.30, and large effect size for *r* of 0.50 and higher according to Cohen’s criteria [54]. Then, regression analyses were conducted to examine the relationship between CEBQ, CFQ and child weight in-depth. Linear regression analyses were performed to estimate the association of eating behavior with the continuous outcome BMI standard deviation scores. Three different models are presented: the first model shows the unadjusted results; the second model is confounder-adjusted; and in the third model the child eating behavior and parental feeding practices are mutually adjusted. Thus, in these analyses the CEBQ-scales are adjusted for the CFQ-scales, while the CFQ-scales are adjusted for the CEBQ-scales. Next, multivariate multinomial logistic regression analyses were conducted to calculate children’s risk for being underweight, overweight or obese as compared to children with a normal weight. Finally, sensitivity analyses were conducted to test the robustness of our findings. In the first sensitivity analysis, we aimed to estimate whether the used cut-off for underweight that resulted in a fairly high percentage of children with underweight influenced our results. We repeated the multinomial logistic regression analyses with underweight defined using stricter cut-offs, i.e. based on a BMI of one or two standard deviations below the mean. In the second part of the sensitivity analyses, to evaluate whether imputation of missing values on child BMI influenced our findings, the linear and multinomial logistic regression analyses were repeated in the subsample of 3157 children with data on BMI available.

Missing values on child BMI (n missings=1828) and the confounders (n missings ranged from 4 in birth weight to 1301 and 1743 for paternal BMI and psychopathology, respectively) were estimated using multiple imputation techniques [55]. All variables included in the multivariate regression analyses as well as available information on child BMI at younger ages were used to estimate missing values [56]. The regression analyses were performed on the imputed datasets (*n* = 4987) and the reported effect estimates are the pooled results of five imputed datasets. All statistical analyses were performed using SPSS version 17.0.

## Results

Characteristics of the children and their parents are presented in Table 1. The majority of the children included in the study population (78*%*) had a normal weight. At age four years, 13*%* of the children had underweight, 8*%* overweight, and 2*%* obesity. Children with underweight, overweight or obesity were more often of non-Dutch origin than children with a normal weight, *χ*^2^(6, 3044) = 58, *p <* .001. No gender differences were found in weight status of the children, *χ*^2^(3, 3072) = 8, *p* = .057. Mothers of children with obesity were more often lower educated, *χ*^2^(1, 2388) = 22, *p* < .001, and had a higher mean BMI, *F*(1, 2229) = 478, *p* < .001, than mothers of children with a normal weight.

**Table 1:** Population characteristics according to the weight status of the children

			**Weight status of children**				
			**Underweight**	**Normal weight**	**Overweight**	**Obese**	
**Child characteristics**		**Total**	***n****=***397**	***n*** = **2473**	***n*** = **239**	***n*** = **48**	***p***^¶^
Gender (% boy)		50.1	49.7	51.2	42.1	45.7	.057
National origin (*%*):	Dutch	66.7	61.0	70.4	57.0	31.1	<.001
	Other Western	9.3	9.7	8.7	10.1	20.0	
	Non-Western	24.0	29.3	20.9	32.9	48.9	
Birth weight (grams)		3444 (567)	3193 (580)	3460 (554)	3657 (535)	3493 (653)	<.001
**Parental characteristics**							
Maternal educational level (*%*)^$^:	High	57.5	54.2	59.5	46.7	24.4	<.001
	Low	42.5	45.8	40.5	53.3	75.6	
Family income (*%*)^$^:	*>*2200 euro	67.3	61.6	70.4	59.7	35.7	<.001
	*<*2200 euro	32.7	38.4	29.6	40.3	64.3	
Smoking during pregnancy (*%*)^$^:	No	78.0	77.8	78.0	74.8	65.0	.185
	Yes	22.0	22.2	22.0	25.2	35.0	
BMI mother (weight/length^2^)		24.4 (4.1)	23.2 (3.6)	24.3 (3.8)	26.5 (5.2)	27.6 (5.6)	<.001
BMI father (weight/length^2^)		25.2 (3.3)	24.8 (3.1)	25.1 (3.2)	26.5 (3.6)	28.3 (3.6)	<.001
Global psychopathology mother (score)		0.13 (0–2.8)	0.13 (0–1.7)	0.13 (0–2.4)	0.15 (0–1.9)	0.19 (0–1.0)	0.228
Global psychopathology father (score)		0.06 (0–3.4)	0.06 (0–1.5)	0.06 (0–2.1)	0.06 (0–0.8)	0.11 (0–0.6)	0.119

Values are percentages for categorical, means (SD) for birth weight and BMI, and medians (100% range) for psychopathology score. ^#^*n* = 3157, as this table represents unimputed data. ^¶^*p* indicates statistical significance of between-group differences. ^$^ Covariates were dichotomized for the purpose of this table only and included in the analyses as follows: education (low, mid-low, mid-high, high), income (*<*1200, 1200–2200, *>*2200 euro’s per month), and smoking (non-smoking, until pregnancy was known, continued smoking).

Table 2 shows that correlations of all CEBQ- and CFQ-scales, except Emotional Overeating, Desire to Drink and Monitoring, with children’s BMI represented small to medium effect sizes. All eating behavior scales were significantly correlated with at least one of the feeding practices scales, although most of these associations were rather small, e.g. Monitoring and Fussiness, *r* = −.038, *p <* .001. Medium effect size correlations were found for Food Responsiveness and Restriction, *r* = .266, *p <* .001, Enjoyment of Food and Pressure to Eat, *r* = −.338, *p <* .001, and for Satiety Responsiveness and Pressure to Eat, *r* = .404, *p <* .001. Correlations between the CEBQ subscales (data not shown) indicated mostly small to medium effect sizes, e.g. Emotional Overeating and Emotional Undereating, *r* = .277, *p <* .001, and large correlations were found between the subscales Saturation Responsiveness, Enjoyment of Food and Fussiness (all *r* > .450, *p <* .001). The CFQ-scales were only weakly correlated with each other (all *r* < .200, *p <* .001).

**Table 2:** Correlations between the CEBQ scales, CFQ scales and child BMI SD scores

	**Pearson correlation coefficients**			
		**Child feeding questionnaire**		
	**BMI SD scores**	**Monitoring**	**Restriction**	**Pressure to Eat**
BMI SD scores	--	-.009	.087 **	-.186 **
Children’s Eating Behaviour Questionnaire				
*Food avoidant*				
Emotional Undereating	-.102 **	.001	.112 **	.160 **
Satiety Responsiveness	-.236 **	-.047 **	.064 **	.404 **
Fussiness	-.079 **	-.038 **	.075 **	.227 **
*Food approach*				
Emotional Overeating	.034	-.144 **	.148 **	.082 **
Food Responsiveness	.219 **	-.029 *	.266 **	-.131 **
Enjoyment of Food	.155 **	.159 **	-.003	-.338 **
Desire to Drink	.017	-.116 **	.110 **	.150 **

** *p <* .001, * *<* .05.

In Table 3, linear associations of CEBQ and CFQ with BMI standard deviation (*SD*) scores are shown. The eating behavior scales Food Responsiveness and Enjoyment of Food were associated with higher BMI SD scores of children. These associations attenuated only slightly and remained highly significant after adjustment for possible confounding factors, such as maternal BMI and indicators of family socioeconomic status. The CEBQ food avoidant scales had a negative relation with children’s BMI SD scores. Again, these associations remained statistically significant in the adjusted analyses. For instance, a one standard deviation higher score on Satiety Responsiveness was associated with a 0.23 lower BMI SD scores, *p <* .001, in the unadjusted analyses, and with a 0.21 lower BMI SD scores, *p <* .001, adjusted for measured confounders. Associations of Emotional Undereating, Satiety Responsiveness, and Food Responsiveness with children’s BMI SD scores attenuated about 20% after adjustment for CFQ-scales, while the parental feeding practices explained 43% of the relation between Fussiness and BMI SD scores (attenuation from B_model 2_ = −0.07 to B_model 3_ = −0.04, see Table 3).

**Table 3:** Association of child eating behavior and eat-related parenting with children’s BMI SD scores

			**BMI standard deviation scores**			
	**Model 1: unadjusted**		**Model 2**^#^		**Model 3**^¶^	
**CEBQ** (**per *SD***)	***B*** (**95***%***CI**)	***p***	***B*** (**95***%***CI**)	***P***	***B*** (**95***%***CI**)	***p***
*Food avoidant*						
Emotional Undereating	−0.10 (−0.13, -0.07)	<.001	−0.08 (−0.10, -0.05)	<.001	−0.06 (−0.09, -0.04)	<.001
Satiety Responsiveness	−0.23 (−0.26, -0.20)	<.001	−0.21 (−0.24, -0.18)	<.001	−0.17 (−0.21, -0.14)	<.001
Fussiness	−0.08 (−0.12, -0.05)	<.001	−0.07 (−0.10, -0.04)	<.001	−0.04 (−0.07, -0.01)	.019
*Food approach*						
Emotional Overeating	0.02 (−0.01, 0.05)	.176	--	--	--	--
Food Responsiveness	0.23 (0.19, 0.26)	<.001	0.21 (0.18, 0.24)	<.001	0.17 (0.13, 0.21)	<.001
Enjoyment of Food	0.14 (0.11, 0.18)	<.001	0.15 (0.11, 0.18)	<.001	0.10 (0.06, 0.14)	<.001
Desire to Drink	0.02 (−0.01, 0.06)	.184	--	--	--	--
**CFQ** (**per *SD***)						
Monitoring	−0.02 (−0.05, 0.02)	.343	--	--	--	--
Restriction	0.09 (0.07, 0.12)	<.001	0.09 (0.07, 0.12)	<.001	0.06 (0.04, 0.09)	<.001
Pressure to Eat	−0.18 (−0.21, -0.15)	<.001	−0.17 (−0.20, -0.14)	<.001	−0.09 (−0.12, -0.06)	<.001

^#^ Model 2: adjusted for child gender, national origin, birth weight, age at questionnaire and BMI assessment, maternal educational level, family income, smoking during pregnancy, and maternal and paternal BMI and psychopathology. ^¶^ Model 3: model 2 additionally adjusted for CFQ-scales (analyses with CEBQ as determinant) or CEBQ-scales (analyses with CFQ as determinant).

Of the CFQ scales, Restriction was positively and Pressure to Eat negatively related with child BMI SD scores. Parental Monitoring was not associated with child BMI. Children’s eating behaviors accounted for a substantial part of the associations between Restriction and child BMI SD scores (33%), and between Pressure to Eat and BMI SD scores (47%), although both scales remained significantly associated with children’s BMI SD scores.

Table 4 presents the relation of CEBQ, CFQ and risk of underweight, overweight and obesity adjusted for measured confounders. The trends show a similar pattern as the linear associations presented in Table 4. For instance, children with higher scores on Food Responsiveness were relatively less often underweight, and more often overweight or obese than children with lower scores, *p* for trend < .001. A one standard deviation higher score on this scale was associated with a more than two-fold risk of being obese (OR = 2.17, 95*%* CI, 1.77-2.65). All assessed CEBQ and CFQ scales, except Emotional Overeating, Desire to Drink, and parental Monitoring were highly associated with children’s weight status.

**Table 4:** Eating behavior and risk of underweight, overweight and obesity

	**OR for weight status of children (95% CI)^#^**				
	**Underweight**	**Normal weight**	**Overweight**	**Obese**	
**CEBQ** (**per *SD***)	***n*** = **645**	***n*** = **3877**	***n*** = **400**	***n*** = **65**	***p *for trend**
*Food avoidant*					
Emotional Undereating	**1**.**10** (1.00-1.22)	Reference	**0**.**76** (0.67-0.85)	**0**.**75** (0.57-0.98)	<.001
Satiety Responsiveness	**1**.**48** (1.34-1.64)	Reference	**0**.**63** (0.56-0.71)	**0**.**43** (0.32-0.57)	<.001
Fussiness	1.10 (0.99-1.22)	Reference	0.92 (0.82-1.04)	0.75 (0.56-1.01)	.001
*Food approach*					
Emotional Overeating	0.97 (0.87-1.08)	Reference	0.99 (0.86-1.15)	0.93 (0.72-1.22)	.865
Food Responsiveness	**0**.**75** (0.65-0.86)	Reference	**1**.**56** (1.41-1.73)	**2**.**17** (1.77-2.65)	<.001
Enjoyment of Food	**0**.**78** (0.69-0.88)	Reference	**1**.**28** (1.13-1.45)	**1**.**74** (1.30-2.33)	<.001
Desire to Drink	1.03 (0.92-1.14)	Reference	0.95 (0.81-1.12)	1.16 (0.90-1.50)	.581
**CFQ** (**per *SD***)					
Monitoring	0.95 (0.86-1.05)	Reference	1.02 (0.90-1.17)	1.01 (0.77-1.31)	.499
Restriction	**0**.**85** (0.78-0.92)	Reference	**1**.**20** (1.06-1.35)	**1**.**46** (1.11-1.91)	<.001
Pressure to Eat	**1**.**30** (1.18-1.43)	Reference	**0**.**66** (0.58-0.74)	**0**.**52** (0.40-0.69)	<.001

^#^ Analyses adjusted for child gender, national origin, birth weight, age at questionnaire and BMI assessment, maternal educational level, family income, smoking during pregnancy, and maternal and paternal BMI and psychopathology.

Sensitivity analyses indicated our findings were fairly robust. First, the results of the logistic regression analyses were largely unchanged when underweight was defined using stricter cut-offs, i.e. based on the lowest BMI decile (10.3% underweight) or on a BMI of two standard deviations below the mean (1.3% underweight). Some of the associations using the more stringent cut-off, however, did not reach statistical significance due to smaller numbers of underweight children (e.g. Food Responsiveness OR = 0.75, 95*%* CI, 0.50-1.12). Second, the linear and multinomial logistic regression analyses were repeated in the subsample of 3157 children with data on BMI available. Again, the results of these analyses were very similar to the results presented in Tables 3 and 4 (e.g. Table 4, Fussiness: p-value for trend 0.006 in unimputed data and 0.001 in imputed data) indicating that imputation of missing values on child BMI hardly influenced our findings.

## Discussion

This large population-based study among four-year-olds showed that young children’s eating patterns and feeding practices of parents are strongly associated with children’s BMI. Not only children with overweight but underweight children also had different eating behaviors than children with a healthy weight. Furthermore, analyses with feeding practices of parents showed a fairly graded association across the whole range from children’s underweight to overweight and obesity. The direction of the reported associations are much in line with previous findings among older children in the primary school-ages [15-18,37,38] suggesting an early age onset of relationships between eating behavior, parental feeding and child BMI. Our observations were largely unaffected by known predictors of unhealthy weight in childhood, such as low socioeconomic background, national origin and parental weight status. As expected, associations between eating behavior and BMI of children attenuated, but persisted after controlling for parental feeding practices. Thus, part of the association between children’s eating behaviors and BMI was due to relations between parental feeding practices and child BMI, suggesting complex associations between these variables. Although we hypothesized that parental feeding practices would be associated with child weight entirely through its effect on child eating behaviors, our results showed that feeding styles were related to offspring BMI even after adjustment for children’s eating behavior. Possibly, parental feeding is associated with child BMI through other dimensions of child eating behavior, such as loss of control or binge eating. However, as a wide range of child eating behaviors was examined, our findings suggest that the behaviors of children and their parents are independently associated with children’s BMI.

Before the results can be discussed, it is essential to consider the reported prevalence rates first. In our study population, approximately one out of ten children was overweight or obese. Although this percentage is lower than the global prevalence estimates of overweight in childhood and adolescence [1-3], it is consistent with the general notion that overweight is somewhat less prevalent among preschoolers as compared with older children [2]. Furthermore, the 2% of children with obesity in our study is very comparable with a recent representative Dutch study reporting obesity prevalence rates of 1% and 3% for 4 year old boys and girls, respectively [57]. However, the reported overweight rates (11%; boys 8%, girls 14%) in this nation-wide study were somewhat higher than those observed in our study (8%). Possibly, children with low socioeconomic background were somewhat underrepresented in our more urban sample, this may account for a slightly lower prevalence of overweight, as children from families with lower socioeconomic status are at risk of overweight [58].

About 13% of the four-year-olds in our study were underweight, which is a higher prevalence estimate than previously shown in school-aged children [4,5]. However, the prevalence rates of underweight are less established than overweight and obesity rates, particularly in early childhood. An alternative explanation for differences in prevalence of underweight might lie in the observation that, especially in girls, the prevalence of underweight is increasing [5].

As hypothesized, children’s food approach behaviors Food Responsiveness and Enjoyment of Food were positively related to children’s BMI. These scales address children’s general appetite for food with Enjoyment of Food measuring normal variation in general appetite, while the Food Responsiveness scale is designed to detect more dysfunctional levels of appetite such as the tendency to continue eating if given the opportunity. It has been suggested that these food approach behaviors become more apparent as children get older and can make more independent choices about food [44], but we observed substantial variability in these traits already in preschoolers. This confirms findings from earlier studies focusing on overweight [15,25,29], and adds to the current literature that underweight children also had relatively low levels on Enjoyment of Food and Food Responsiveness. These relations between appetite and the BMI spectrum might be explained by genetic variants contributing to both children’s weight status and their susceptibility to eating in response to the presence of foods [59]. Genes can exert a direct influence on child behavior and weight status, but can also work indirectly through the early food environment that is primarily provided by the parents. For instance, overweight parents might provide an obesogenic eating environment which stimulates appetite and food intake in the offspring, while relatively lean parents may discourage the overconsumption of food. However, behavioral genetics of child eating patterns are relatively understudied and future research is needed to clarify the interplay between genetic and environmental influences on children’s eating and weight development [59].

In contrast to what we hypothesized, we found no evidence that the food approach scales Desire to Drink or Emotional Overeating were associated with child BMI. The findings with Desire to Drink replicates results from previous studies for which the authors argued that the lack of finding an association between drinking and weight might have been due to limited statistical power [26,36]. Apparently, being a thirsty person or the amount of drinking per se is not related with child BMI. The type of beverages, i.e. the consumption of high-energy drinks, probably has more influence on weight status [60]. Regarding eating in response to emotional cues, it has been suggested that the food approach scale Emotional Overeating reflects the opposite of the food avoidant Emotional Undereating [46]. However, in our study, we found a positive correlation between the two scales. Moreover, we showed an association between emotional undereating and BMI, while surprisingly, emotional overeating was not related with weight status in these very young children. Emotional distress may lead to inhibition of appetite, but does not result in food craving in young children, suggesting that these emotional eating behaviors cannot be simply seen as two extremes of the same continuum. Alternatively, the children in the present study might have been too young to exhibit excessive eating and snacking, as they probably do not have free access to foods yet. This hypothesis is substantiated by previous studies showing that increasing BMI was associated with progressively higher levels of emotional overeating among school-aged children [16,38,46].

In line with our hypotheses, not only Emotional Undereating, but the food avoidant scales Satiety Responsiveness and Fussiness were also associated with progressively lower weights in children. This finding for Fussiness contrasts with previous studies [25,35,36]. However, in a population-based sample of 1498 Canadian preschoolers, Dubois and colleagues also reported that picky eaters were more likely to be underweight [34]. Our findings and this Canadian study suggest that fussiness is indeed associated with a relatively low BMI in the general population. Possibly, underweight of children leads to higher levels of fussiness, for instance through an adverse effect of control or pressure of parents on children’s eating. However, the analyses substantiate this reasoning only to some extent, as the relation between fussiness and BMI attenuated but remained statistically significant after adjusting for parental pressure to eat and monitoring. Thus, it seems likely that at least part of the association is from pickiness leading to insufficient food intake, which eventually hinders adequate weight gain and growth. Although the CEBQ refers to fussiness about food in general, it is also possible that the food intake of picky children is not diverse enough and lacks essential nutrients like vitamins, minerals, proteins and fibres. Clearly, parents and primary health care professionals should carefully monitor fussy children and their food intake, although causal directions have to be ascertained in longitudinal studies.

A negative association between satiety response and BMI in preschoolers was reported once before [15]. We extend this previous study by showing that both children with overweight and children with underweight have a different satiety response than children with a normal weight. Possibly, some young children have a suboptimal down regulation of their food consumption resulting in excessive weight gain, while other children have a too effective satiety response resulting in underweight. However, that parental feeding practices accounted for part of this association suggests more complex pathways. Parental pressure to eat might reflect a mediation effect, as toddlers who are highly responsive to internal satiety cues and quickly feel full might be pressured by their parents to eat more, which can be counterproductive and actually result in *less* eating [19]. Alternatively, restrictions of parents regarding food intake could result in a poor responsiveness to internal hunger and satiety cues, thereby influencing children’s food intake and weight gain.

As hypothesized, restrictive parenting during mealtimes was positively and parental pressure to eat was negatively associated with children’s BMI. Within the framework of a child-responsive model, these feeding strategies can be interpreted as a response to child weight: parental efforts to restrict food intake may be a response to children’s overweight, while parents of children with underweight probably pressure their children to eat more. However, the observed associations are probably more complex. It is not possible to infer causality from our cross-sectional study, but the findings suggest a number of explanations. Parental restriction was correlated to children’s Food Responsiveness, and child eating behavior attenuated the association between restriction and child BMI. This suggests that restrictive parenting might stimulate poor intake regulation and overeating at times when access to food is not restricted, eventually resulting in weight gain. Parental pressure to eat may be associated with child weight through a counterproductive effect of lowering children’s enjoyment of food, eventually resulting in eating less and weight loss. Alternatively, pressure to eat might also be a parent’s response to children quickly feeling ‘full’. This explanation is substantiated by the correlation between Pressure to Eat and CEBQ Satiety Responsiveness. Clearly, longitudinal research with repeated measurements of children’s eating behavior, feeding practices of parents and child BMI is needed to further unravel these pathways.

We found no evidence that parental monitoring of children’s food intake is associated with child BMI, which is in line with previous studies using small convenience samples [25,26,28,30-33]. Perhaps, keeping track of the amount of sweets, snacks and high-fat food children consume is a very common behavior of parents, not necessarily related to children’s BMI. Alternatively, parents might have provided socially desirable answers on the Monitoring-items.

Some limitations of this study have to be discussed. Firstly, a number of children had no information on eating behavior or BMI. While missing data on BMI was rather random, information on eating behavior was more complete in Dutch children of relatively high educated mothers. However, although this selective response may have influenced the reported prevalence estimates, it probably has had less effect on the associations reported in our study [61]. Secondly, although children’s anthropometrics were measured objectively, assessments of problematic eating behaviors were based on mothers’ subjective opinions of children’s behavior. Even though the analyses were adjusted for several maternal characteristics, it cannot be ruled out completely that a mother’s well-being and her attitudes about health influenced her ratings of children’s eating behavior. On the other hand, validation studies indicated that parent reports of children’s eating behaviors, such as the CEBQ and CFQ, correlated substantially with children’s actual food intake [34,62]. Another limitation is the study’s cross-sectional design which precludes inferences about causation. Longitudinal studies are essential to detect whether feeding practices of parents and children’s eating behaviors predict the development of weight problems, or if they are associated concurrently only.

## Conclusions

In a large population-based sample, we showed that food responsiveness, enjoyment of food and parental restriction are associated with progressively higher weights of children. The findings regarding these food approach behaviors of children are especially worrisome in the context of the current, rather obesogenic environment [63], as such settings place children who are interested in food at risk for developing overweight. Furthermore, our study showed that children with underweight have distinct eating behaviors and that their parents are more likely to pressure during mealtimes. The associations between preschoolers’ eating behavior, parental feeding patterns and child BMI are complex and need further investigation. Meanwhile, health care professionals should be aware that there is a complex interplay between children and their parents regarding eating, and that the behaviors of both children and their parents are to some extent independently associated with child BMI. This implies that if behaviors are indeed causally related to child BMI – which seems quite plausible – efforts to prevent or treat unhealthy child weight might benefit from a focus on changing the behaviors of both children and their parents.

The high prevalence of underweight warrants awareness of health care practitioners who – in the current obesity epidemic – may be more focused on detecting and treating overweight than underweight. The incessant nature and long-term health consequences of childhood weight problems [6-10] and the observed high prevalence of under- and overweight among four-year-old children suggests preventions and interventions targeting unhealthy weights should start early in life. Longitudinal studies are needed for causal inferences. Yet, it is tempting to speculate that assessing eating and feeding patterns at a young age – for instance at child health centers – could help identify children at risk for over- and underweight, as these behaviors are already highly associated with children’s BMI by the age of four years. The effectiveness of such a screening policy should be carefully monitored and this practice shift should be evaluated in terms of costs and benefits.

## Abbreviations

BMI: Body mass index; CEBQ: Child eating behaviour questionnaire; CFQ: Child Feeding Questionnaire; OR: Odds ratio; SD: Standard deviation; 95*%* CI: 95*%* confidence interval.

## Competing interests

The authors declare that they have no competing interests.

## Authors’ contributions

PWJ conceptualized the study, performed statistical analyses and drafted the manuscript. SJR made substantial contributions to the acquisition and interpretation of the data. JDM was also involved in the interpretation of the data. VWVJ, HR, AH, and FCV made substantial contributions to the conception and design of the study. VWVJ, AH and FCV also acquired funding for the study. HT was involved in the conceptualization of the study, the interpretation of the data and supervised the drafting of the manuscript. All authors critically revised the manuscript and approved the final version of the manuscript.
